# Fat mass and obesity associated (*FTO*) gene influences skeletal muscle phenotypes in non-resistance trained males and elite rugby playing position

**DOI:** 10.1186/s12863-017-0470-1

**Published:** 2017-01-19

**Authors:** S. M. Heffernan, G. K. Stebbings, L. P. Kilduff, R. M. Erskine, S. H. Day, C. I. Morse, J. S. McPhee, C. J. Cook, B. Vance, W. J. Ribbans, S. M. Raleigh, C. Roberts, M. A. Bennett, G. Wang, M. Collins, Y. P. Pitsiladis, A. G. Williams

**Affiliations:** 1 MMU Sports Genomics Laboratory, Manchester Metropolitan University, Crewe Green Road, Crewe, CW1 5DU UK; 2 A-STEM, College of Engineering, Swansea University, Swansea, UK; 3 Research Institute for Sport & Exercise Sciences, Liverpool John Moores University, Liverpool, UK; 4 Institute of Sport, Exercise and Health, University College London, London, UK; 5 Centre for Physical Activity and Chronic Disease, Institute of Health and Wellbeing, University of Northampton, Northampton, UK; 6 School of Sport, Health and Exercise Sciences, Bangor University, Bangor, UK; 7 Institute of Cardiovascular & Medical Sciences University of Glasgow, Glasgow, UK; 8 Medical and Scientific Department, South African Rugby Union, Cape Town, South Africa; 9 Discipline of Sports Science, Faculty of Health Sciences, University of Kwazulu-Natal, Durban, South Africa; 10 FIMS Reference Collaborating Centre of Sports Medicine for Anti-Doping Research, University of Brighton, Brighton, UK; 11 Division of Exercise Science and Sports Medicine, Department of Human Biology, University of Cape Town (UCT), Cape Town, South Africa; 12 School of Healthcare Science, Manchester Metropolitan University, Manchester, UK

**Keywords:** RugbyGene project, IRX3, Lean mass

## Abstract

**Background:**

*FTO* gene variants have been associated with obesity phenotypes in sedentary and obese populations, but rarely with skeletal muscle and elite athlete phenotypes.

**Methods:**

In 1089 participants, comprising 530 elite rugby athletes and 559 non-athletes, DNA was collected and genotyped for the *FTO* rs9939609 variant using real-time PCR. In a subgroup of non-resistance trained individuals (NT; *n* = 120), we also assessed structural and functional skeletal muscle phenotypes using dual energy x-ray absorptiometry, ultrasound and isokinetic dynamometry. In a subgroup of rugby athletes (*n* = 77), we assessed muscle power during a countermovement jump.

**Results:**

In NT, TT genotype and T allele carriers had greater total body (4.8% and 4.1%) and total appendicular lean mass (LM; 3.0% and 2.1%) compared to AA genotype, with greater arm LM (0.8%) in T allele carriers and leg LM (2.1%) for TT, compared to AA genotype. Furthermore, the T allele was more common (94%) in selected elite rugby union athletes (back three and centre players) who are most reliant on LM rather than total body mass for success, compared to other rugby athletes (82%; *P* = 0.01, OR = 3.34) and controls (84%; *P* = 0.03, OR = 2.88). Accordingly, these athletes had greater peak power relative to body mass than other rugby athletes (14%; *P* = 2 x 10^-6^).

**Conclusion:**

Collectively, these results suggest that the T allele is associated with increased LM and elite athletic success. This has implications for athletic populations, as well as conditions characterised by low LM such as sarcopenia and cachexia.

## Background

Fat mass and obesity associated (*FTO*) is the most investigated gene in obesity and has complex molecular mechanisms that are yet to be elucidated. Recent genome-wide association studies (GWAS) have identified several common single nucleotide polymorphisms (SNP) in the human *FTO* gene associated with obesity, body mass index BMI; [1], cardiovascular disease and hypertension [2, 3]. These *FTO* SNPs, which are in strong linkage disequilibrium (*r* ^*2*^ > 0.80), are located in a cluster on the first intron of the gene on chromosome 16 and consequently exhibit similar obesity-related traits [4]. Thus, within different *FTO* variants, those alleles that have been positively associated with obesity-related phenotypes are referred to as risk alleles, while those negatively associated with such traits are referred to as protective alleles. Homozygotes for the minor risk allele consistently demonstrate greater BMI and body mass (3-10 kg) in comparison to protective allele carriers [5, 6]. This greater body mass is likely to be adipose tissue [7–11], although there exist some suggestions of greater lean mass (LM) in addition to fat mass [9, 12] and independent of fat intake and physical activity [9]. This suggests that *FTO* genotype may be related to muscle properties and is supported by evidence from a large UK twin study that related *FTO* SNPs with body composition while controlling for lean mass and fat mass (separately and combined). The authors concluded that *FTO* SNP associations with body size were a composite of both lean and fat mass, not fat mass alone [13].

Environmental lifestyle factors (diet and physical activity) have also been investigated for *FTO* gene-environment interactions. Risk allele carriers are more likely to choose a high fat diet than protective allele carriers [11, 14, 15]. However, with administration of a high protein diet (25% energy intake) risk allele carriers demonstrated a greater reduction in body mass, fat mass and percentage body fat [16], due to greater appetite suppression than in protective allele carriers [17]. Additionally, physically active risk allele carriers have a 30% reduction in likelihood of becoming obese and have 36% less body fat compared to inactive risk allele carrying individuals [18]. In contrast, data from the HERITAGE Family Study showed that following 20 weeks of endurance training, protective allele carriers exhibited reductions in fat mass three times greater than risk allele homozygotes [19]. Interestingly, when comparing normal weight and obese individuals who participate in sport, no differences in *FTO* genotype were observed (*P* = 0.97), which was contrasted by those not participating *P* = 0.02; [20]. Considering the attenuation of *FTO-*associated obesity with environmental factors and the greater *FTO-*associated LM reported in obese populations [9, 12], investigating LM and associated phenotypes in healthy, non-obese, non-resistance trained individuals and habitually trained elite athletes would be worthwhile.

To date, there have been no investigations of in vivo skeletal muscle phenotypes in trained or non-resistance trained populations for associations with *FTO* genotype. Eynon et al. [21] investigated *FTO* rs9939609 in three European cohorts of power (*n* = 258; 58.3% elite) and endurance athletes (*n* = 266; 57.1% elite) from a variety of sporting disciplines - but identified no associations. This lack of association was likely due to the considerable differences in physiological demand between the various athletic disciplines included, plus further variability in the standard of athlete. We have recently shown the ability of genetic research in a single sport with player roles that differ distinctly, namely rugby union (RU), to reveal context-specific competitive advantages provided by particular alleles [22]. Therefore, as RU includes athletes of remarkably distinct anthropometric and body composition phenotypes, elite RU provides a unique opportunity to investigate *FTO* in individuals at the extreme upper end of physical fitness [23].

Therefore, the main aims of the present study were to (1) investigate any association of *FTO* rs9939609 with body composition and muscle structural and functional parameters in a homogenous, healthy, non-obese non-resistance trained population (2) investigate whether *FTO* rs9939609 genotype differed between elite rugby athletes and a control population, and/or between RU player positions. Based on prior data in obese populations, it was hypothesised that the rs9939609 risk (A) allele would be associated with greater body mass, fat mass, BMI, LM, muscle volume and muscle strength in non-resistance trained individuals. Secondly, for the elite rugby cohort, it was hypothesised that the *FTO* A allele would be overrepresented in player positions typically requiring greater body and muscle mass while the protective (T) allele would be more common in positions requiring a lean phenotype.

## Method

### Participants

A total of 1089 individuals were recruited and gave written informed consent to participate in the present study. The total sample comprised elite Caucasian male rugby athletes (*n* = 530; height 1.85 (0.07) m, mass 101 (14) kg, age 29 (7) yr, BMI 29.4 (3.7) kg∙m^-2^; mean (standard deviation (SD)) including 73% British, 16% South African, 7% Irish and 4% from other nationalities and non-athlete Caucasian control participants (male and female; *n* = 559; height 1.75 (0.10) m, mass 75 (13) kg, age 29 (16) yr, BMI 24.5 (3.6) kg∙m^-2^) including 86% British, 12% South African, 1% Irish and 1% from other nationalities. Athletes were considered elite if they had competed regularly (> 5 matches) since 1995 in the highest professional league in the UK, Ireland or South Africa for RU or the highest professional league in the UK for rugby league (RL). Of the RU athletes, 52.7% had competed at an international level for a “High Performance Union” (Regulation 16, worldrugby.org) and 43.2% of RL had competed at international level. All data for the athlete group’s international status were confirmed as of 1st June 2016. Furthermore, within the rugby cohort, a subsample (*n* = 77) were examined for performance-related muscle phenotypes. Within the control group were a subgroup of non-resistance trained healthy males (NT; Table 1). NT participants were aged 18–39 years, had a BMI 18.5–30 kg∙m^-2^, had not undertaken any structured resistance training in the preceding 12 months and had no history of neurological or musculoskeletal disorders. Additionally, only those participants undertaking less than 3 h of low-to-moderate physical activity per week, assessed via questionnaire [24], were included.

**Table 1:** Descriptive, morphological and functional characteristics of all participants and genetic frequency, in the non-resistance trained (NT) cohort

Phenotype	All (*n* = 120)	AA (*n* = 18)	AT (*n* = 58)	TT (*n* = 44)	TT + AT (*n* = 102)	*P* values Additive (Recessive)
Height (m)	1.79 (0.06)	1.80 (0.06)	1.78 (0.07)	1.80 (0.06)	1.79 (0.07)	0.15 (0.48)
Mass (kg)	75.0 (10.0)	81.0 (8.1)	74.2 (10.3)	73.5 (9.6)	73.9 (10.0)	0.03 (0.02)
BMI (kg∙m^-2^)	23.4 (2.7)	25.1 (2.6)	23.5 (2.8)	22.6 (2.4)	23.1 (2.6)	0.02 (0.02)
Age (years)	20.6 (2.3)	21.6 (2.8)	20.9 (2.4)	19.7 (1.6)	20.4 (2.1)	0.02 (0.08)
Fat mass (%)	21.5 (5.2)	23.3 (5.5)	21.8 (5.2)	20.3 (4.8)	21.2 (5.1)	0.09 (0.11)
LM (%)	73.5 (5.9)	70.3 (6.3)	73.3 (5.6)	75.1 (5.8)	74.4 (5.6)	0.04 (0.04)
Total appendicular LM (%)	33.4 (3.8)	31.6 (3.5)	33.0 (3.7)	34.6 (3.7)	33.7 (3.7)	0.04 (0.05)
Arm LM (%)	8.5 (1.2)	7.9 (1.0)	8.6 (1.3)	8.7 (1.1)	8.7 (1.2)	0.06 (0.04)
Leg LM (%)	24.8 (2.9)	23.7 (3.1)	24.4 (2.8)	25.8 (2.9)	25.0 (2.9)	0.04 (0.10)
*V*_VL_ (cm^3^)	566 (86)	585 (81)	550 (86)	580 (85)	563 (86)	0.20 (0.61)
VL ACSA (cm^2^)	21.4 (2.5)	21.9 (2.8)	21.0 (2.4)	21.9 (2.5)	21.4 (2.5)	0.20 (0.86)
VL PCSA (cm^2^)	71.7 (13.9)	71.2 (13.9)	72.0 (13.7)	71.5 (11.0)	71.8 (12.6)	0.97 (0.61)
Isometric MVC_KE_ torque (N∙m)	272 (53)	285 (38)	271 (63)	268 (45)	270 (56)	0.50 (0.25)
VL specific force (N∙cm^-2^)	21.6 (2.6)	22.3 (2.5)	21.6 (2.5)	21.1 (2.8)	21.4 (2.6)	0.50 (0.25)

Data are mean (SD)

*BMI*, body mass index, *LM* lean mass, *VL* vastus lateralis, *ACSA* anatomical cross sectional area, *PCSA* physiological cross-sectional area, *MVC*_*KE*_ maximal voluntary contraction, *V*_*VL*_ vastus lateralis muscle volume

#### Procedures

### Muscle properties in NT

An isokinetic dynamometer (Cybex Norm, Cybex International Inc., NY, USA) was used to assess maximal isometric knee extension (MVC_KE_) and maximal isometric knee flexion (MVC_KF_) torque at knee joint angles of 70°, 80° and 90° (full knee extension = 0°). The angle of peak torque was taken as the optimal knee joint angle and used to estimate antagonist muscle co-activation during MVC, which assumed a linear relationship between biceps femoris EMG activity and knee flexion torque [25]. Together with antagonist co-activation, quadriceps femoris (QF) voluntary activation capacity, determined using the interpolated twitch technique [26], allowed for the calculation of net MVC_KE_ torque. Subsequently, patella tendon moment arm length (*d*_PT_) was measured using dual energy x-ray absorptiometry DXA; [27] and patella tendon force calculated as net MVC_KE_ torque/*d*_PT_. The contribution of the vastus lateralis (VL) muscle (MF_VL_) to patella tendon force was calculated by estimating the relative physiological cross-sectional area (PCSA) of the VL as ~21% of the QF [28]. VL muscle architecture (fascicle length, *L*_f_ and pennation angle, *θ*) was measured at 50% of VL length during MVC_KE_ at the optimal knee joint angle using ultrasound (AU5, Esaote, Italy) and VL fascicle force estimated as MF_VL_/cosine *θ* [29]. At rest, ultrasound was also used to obtain a series of transverse plane scans at 50% of VL muscle length from the medial to lateral borders, which were contour matched to determine VL anatomical cross-sectional area [ACSA; 29]. With VL length and VL ACSA used to estimate VL volume (*V*_VL_) as previously [30]. Subsequently, VL PCSA was calculated as *V*_VL_/*L*_f_ and, VL specific force calculated by dividing VL fascicle force by VL PCSA [29]. Finally, quantification of whole body and appendicular LM was completed using DXA (Hologic Discovery: Vertec Scientific Ltd, UK) following a 12 h overnight fast. Participants lay in a supine position, avoiding any contact between the trunk and the appendicular mass during a 7 min whole body scanning procedure (effective dose, 8.4 μSv). Appendicular lean mass was estimated from the DEXA as the total muscle mass of both the upper and lower limbs, where LM is body mass excluding fat and bone mass.

### Muscle power in RU athletes

Using a portable force platform (Type 92866AA, Kistler, Germany) peak power output (PPO) was determined during a bilateral countermovement jump (CMJ) according to methods described previously [31]. Body mass and the vertical component of the ground reaction force during the CMJ (sampled at 1000 Hz) were used to determine instantaneous velocity and displacement of the participant’s centre of gravity. Instantaneous power output was determined using the following equation: Power (W) = vertical GRF (N) x vertical velocity of centre of gravity (m · s^-1^), with the highest value produced deemed the PPO.

### Sample collection and genotyping

Description of all molecular procedures have previously been described in detail [22]. Briefly, blood (~70% of all samples), saliva (~25%) or buccal swab samples (~5%) were obtained via the following protocols. Blood was drawn from a superficial forearm vein into an EDTA tube and stored in sterile tubes at -20**°**C until processing. Saliva samples were collected into Oragene DNA OG-500 collection tubes (DNA Genotek Inc., Ontario, Canada) according to the manufacturer’s protocol and stored at room temperature until processing. Sterile buccal swabs (Omni swab, Whatman, Springfield Mill, UK) were rubbed against the buccal mucosa of the cheek for approximately 30 s. Tips were ejected into sterile tubes and stored at -20**°**C until processing. DNA isolation and genotyping were performed in the MMU, University of Glasgow, University of Cape Town (DNA isolation only) and University of Northampton laboratories. The majority of samples were processed and genotyped in the MMU laboratory, including all samples within NT. At MMU and Glasgow, DNA isolation was performed using the QIAamp DNA Blood Mini kit and standard spin column protocol, following the manufacturer’s instructions (Qiagen, West Sussex, UK). Briefly, 200 *μL* of whole blood/saliva, or one buccal swab, was lysed, incubated, the DNA washed and the eluate containing isolated DNA stored at 4**°**C. In Cape Town, DNA was isolated from whole blood using a different protocol [32] during which samples were lysed, centrifuged, the DNA washed and samples stored at -20**°**C. Genotyping of DNA isolated in Cape Town was performed in Glasgow. At Northampton, DNA was isolated from whole blood using Flexigene kits (Qiagen). Briefly, samples were lysed, DNA precipitated and washed, with samples stored at -20**°**C.

Genotyping in all three genotyping centres was performed on *FTO* (rs9939609). Briefly, in the Glasgow laboratory 10 *μL* Genotyping Master Mix (Applied Biosystems, Paisley, UK), 1 *μL* SNP-specific TaqMan assay (Applied Biosystems), 6 *μL* nuclease-free H_2_O and 3 *μL* DNA solution (~9 ng DNA) were added per well. In the Northampton laboratory, genotyping was performed by combining 10 *μL* of Genotyping Master Mix, 8 *μL* H_2_O, 1 *μL* assay mix with 1 *μL* of purified DNA (~10 ng). In both laboratories, PCR was performed using a StepOnePlus real-time detector (Applied Biosystems). Briefly, after an initial 10 min at 95°C, 40 cycles of denaturation at 92°C for 15 s then annealing and extension at 60°C for 1 min were used. Genotyping calls were performed using StepOnePlus software version 2.3 (Applied Biosystems). At MMU, 5 *μL* Genotyping Master Mix, 4.3 *μL* H_2_O, 0.5 *μL* assay mix and 0.2 *μL* of purified DNA (~9 ng) were used in each reaction for samples derived from blood and saliva. For DNA derived from buccal swabs, 5 *μL* Genotyping Master Mix was combined with 3.5 *μL* H_2_O, 0.5 *μL* assay mix and 1 *μL* DNA solution (~9 ng DNA). Either a Chromo4 (Bio-Rad, Hertfordshire, UK) or StepOnePlus real-time PCR system was used. Briefly, after an initial 10 min at 95°C, 40 cycles of denaturation at 92°C for 15 s then annealing and extension at 60°C for 1 min were used. Genotyping calls were performed using Opticon Monitor software version 3.1 (Bio-Rad) or StepOnePlus software version 2.3. The Taqman assay included VIC and FAM dyes that indicated A and T alleles on the forward DNA strand, respectively. Thus, VIC/FAM were interpreted as: 5′-GTGAATTT[A/T] GTGATGCA-3′.

### RU positional groups

As established previously [22], to compare genotype and allele frequencies within the RU group, athletes were allocated to subgroups; forwards (props, hookers, locks, flankers, number eights) and backs (scrum halves, fly halves, centres, wings, full backs). Also, due to diverse physiological demands within RU [33, 34], athletes were further divided into established positional groups according to their movement patterns [33]; front five (props, hookers, locks), back row (flankers, number eights), half backs (scrum halves, fly halves), back three (wings and full backs) and centres. For example, in one study the front five travelled ~136 m at  > 5 m · s^–1^ compared to ~566 m for the back three [35]. Comparisons between positions were not performed for the RL cohort due to low statistical power when it was subdivided.

### Data analysis

SPSS for Windows version 22 (SPSS Inc., Chicago, IL) software was used to conduct statistical analyses. One-way analysis of variance (ANOVA) was used to compare height, mass, BMI, age and PPO between sample populations and genotype groups. When genotype groups were compared using a recessive model, an independent samples t-test was used. Furthermore, genotype effects on muscle phenotypes of interest were assessed for linear trend. Pearson’s χ^2^ tests compared genotype and allelic frequencies between athlete and control groups and between RU positional subgroups. There were 30 comparisons for genotype frequency between groups and 28 tests of genotype differences in phenotype in NT that were subjected to Benjamini-Hochberg corrections to control false discovery rate and corrected probability values are reported [36]. Where appropriate, odds ratio (OR) and eta squared (η_P_^2^) were calculated to estimate effect size. Alpha was set at 0.05.

## Results

Genotype calling was successful in all samples. There was 100% agreement among reference samples genotyped in the three genotyping centres, i.e. Glasgow, Northampton and MMU laboratories. Genotype frequencies were in Hardy-Weinberg equilibrium for the entire control group (*P* = 0.871), NT (*P* = 0.988) and athlete groups (RL, *P* = 0.183; RU, *P* = 0.076).

### Non-resistance trained (NT)

The AA genotype group had greater body mass, BMI and age but not height compared to other genotype groups (Table 1). There were genotype differences for total body (η_P_^2^ = 0.072), total appendicular (η_P_^2^ = 0.075) and leg (η_P_^2^ = 0.078) LM, with tendencies for arm LM (*P* = 0.06, η_P_^2^ = 0.054) and total fat mass (*P* = 0.09, η_P_^2^ = 0.024). T-allele carriers demonstrated greater total body (5.3%), appendicular (6.7%) and arm (9.8%), but not leg (*P* = 0.10) LM than AA homozygotes. There were no differences in muscle size, torque or specific force variables (Table 1).

### Athletes

Athletes were taller and heavier (*P* < 0.05) but not older (*P* > 0.05) than controls. There were no genotype frequency differences between athletes (RL and RU combined; *P* = 0.16), RL (*P* = 0.36), RU (*P* = 0.16) and controls (only additive models presented).

**Fig. 1:**
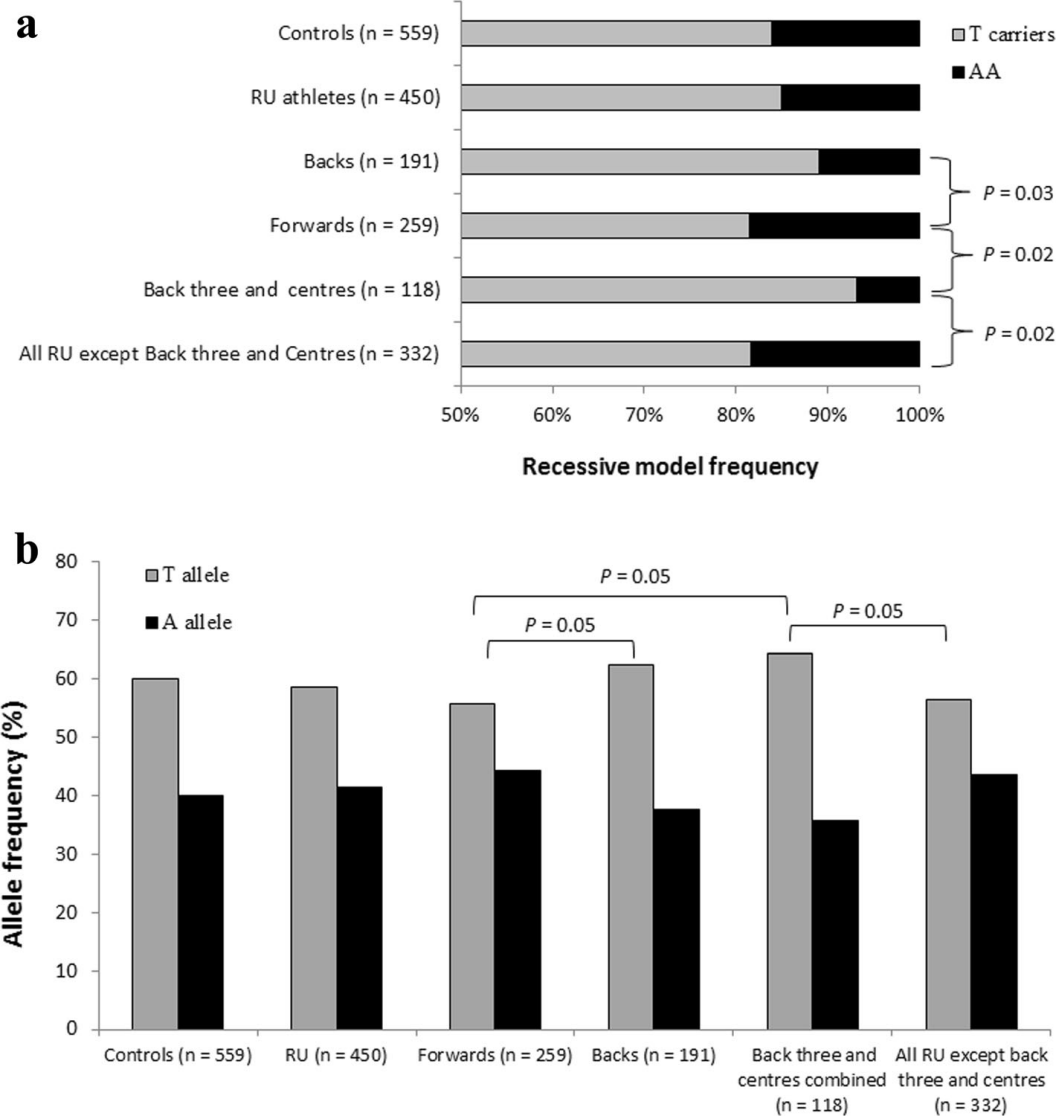
*FTO* genotype data of athletes and controls. **a** Recessive model. *Grey bars* are T allele carriers, *black bars* are AA genotypes. **b** Allele frequency for selected subgroups. *Grey bars* represent the T allele, *black bars* represent the A allele

In terms of player position, backs had a greater frequency of T allele carriers than forwards (*P* = 0.03, Table 2, Fig. 1) and showed greater odds of being T allele carriers than AA genotype (OR = 1.84, Table 3). When combined, the back three and centres group contained less AA homozygotes and more T allele carriers (*P* = 0.03, *P* = 0.02, respectively; Fig. 1a and Table 2) than controls. Additionally, controls had more than twice the odds of being AA than the back three and centres group, with greater odds of T allele carriers in the back three and centres than controls (Table 3). Compared to forwards and all other RU athletes, TT genotype (*P* = 0.03; *P* = 0.03, respectively) and T allele carriers (*P* = 0.02; *P* = 0.02, respectively) were more common in the back three and centres group (Fig. 1a and Table 2). Likewise, forwards and all other RU athletes had greater than three times the odds of being AA genotype than the back three and centres group, with greater odds of T allele carriers in the back three and centres group than forwards and all other RU athletes (Table 3). Furthermore, the back three and centres group showed a greater T allele frequency than both forwards and all other RU athletes (Fig. 1b) and almost one and a half times greater odds of possessing the T allele (Table 3).

**Table 2:** Genotype and allele distribution of controls and athletes separated by code (RL and RU) and into positional subgroups for RU, presented as genotype/allele counts followed by percentage in parentheses

Genotype/allele	All athletes	RL athletes	RU athletes	Controls	Forwards	Backs	Front 5	Back row	Half backs	Back 3 and centres
*FTO*										
AA	80 (15.1)	12 (13.6)	69 (15.3)	90 (16.1)	48 (18.5)	21 (11.0)	30 (17.0)	18 (21.7)	13 (17.8)	8 (6.8)*
AT	280 (52.7)	49 (55.7)	235 (52.3)	266 (47.6)	133 (51.4)	102 (54.7)	94 (53.4)	39 (47.0)	34 (46.6)	68 (57.6)
TT	170 (32.2)	27 (30.7)	146 (32.4)	203 (36.3)	78 (30.1)	68 (34.3)	52 (29.6)	26 (31.3)	26 (35.6)	42 (35.6)^‡^
Total	530	88	450	559	259	191	176	83	73	118
A allele	440 (41.5)	73 (41.5)	375 (41.4)	446 (39.9)	229 (44.2)	144 (37.7)	154 (43.7)	75 (45.2)	60 (41.1)	84 (35.6)^‡^
T allele	620 (58.5)	103 (58.5)	527 (58.6)	672 (60.1)	289 (55.8)	238 (62.3)	198 (56.3)	91 (54.8)	86 (58.9)	152 (64.4)

Eight athletes competed in both elite RL and RU and were included in both groups that were analysed separately. *Different from controls (*P* < 0.04). ^**‡**^Different from forwards (*P* = 0.03)

**Table 3:** Odds Ratio statistics for RU athlete status by playing position for *FTO* genotype (TT/AA), allele (T/A) and recessive (T/AA) genetic models

Positional comparison	Genetic model	Odds ratio	95% Confidence interval	*P* value
Backs v Forwards	T/AA	1.84	1.06-3.19	0.029
Back 3 and centres v Controls	TT/AA	2.33	1.05-5.16	0.038
	T/AA	2.64	1.05-5.16	0.012
Back 3 and centres v Forwards	TT/AA	3.23	1.39-7.46	0.006
	T/AA	3.12	1.43-6.84	0.004
	T/A	1.44	1.04-1.97	0.026
Back 3 and centres v all other RU athletes	TT/AA	3.08	1.36-6.98	0.007
	T/AA	3.09	1.43-6.68	0.004
	T/A	1.37	1.01-1.86	0.045
Back 3 and centres v other backs	TT/AA	2.98	1.17-7.59	0.022
	T/AA	2.63	0.96-7.19	0.060

### Muscle power

While PPO tended to be greater in back row players with lowest power in the halfbacks (5792 vs 5000 W; *P* = 0.09), PPO relative to body mass did differ according to playing position (Table 4). The centre and back 3 players (59 W∙kg^-1^) were 9.8% more powerful than back row (54 W∙kg^-1^; *P* = 0.025) and 20.2% more powerful than front 5 players (47 W∙kg^-1^; *P* = 6 x 10^-7^; Table 4).

**Table 4:** Muscle power of RU athletes (*n* = 77) in positional groups. *P * values are from comparisons of power between the four groups. Data are mean (SD)

Phenotype	Front 5 (*n* = 32)	Back row (*n* = 14)	Half backs (*n* = 14)	Back 3 and centres (*n* = 17)	*P* value
Power (W)	5592 (819)	5687 (858)	4937 (650)	5579 (569)	0.030
Relative Power (W∙kg^-1^)	49.4 (7.7)	52.7 (6.9)	56.1 (7.2)	59.8 (4.2)*	2 x 10^-5^

*Different from all other players (*P* = 8 x 10^-7^), including front 5 (*P* = 3 x 10^-6^) and back row (*P* = 0.005)

## Discussion

We have shown that individuals possessing the *FTO* rs9939609 T allele and TT genotype had greater LM, while no differences in leg muscle size or strength were observed (Table 1), thus rejecting our first hypothesis that the risk (A) allele would be associated with greater LM and muscle volume in a healthy non-resistance trained population. That greater LM in T allele and TT genotype individuals was observed despite A allele carriers having greater body mass and BMI as reported previously [5, 6]. In agreement with our second hypothesis, we report a greater T allele and TT genotype frequency in elite rugby athlete playing positions more reliant on a lean phenotype for success [37], while the A allele is more common in those positions where total body mass is more important [38]; Fig. 1, Tables 2 and 3. The ability to rapidly produce high levels of power relative to body mass using the leg musculature was greater in those playing positions more reliant on a lean phenotype (Table 4) and is in agreement with previously published data of elite RU players [39]. One possible biological mechanism underlying the present results may be the action of the iroquois homeobox 3 (IRX3) protein through its *FTO* genomic loci interaction.

Until recently, little was known about the molecular basis for *FTO* SNP associations with any reported phenotype measure, because there was no association between *FTO* SNPs and expression of the *FTO* protein [40, 41]. However, *FTO* has recently been found to influence IRX3 protein expression, through evolutionarily conserved long-range chromatin looping. Individuals possessing the protective *FTO* genotype/allele (TT/T) display lower IRX3 expression than AA homozygotes [42]. Furthermore, in contrast to IRX3 knockout (KO) mice, wild type mice exhibited similar *FTO* SNP risk (A) allele-associated phenotypes, such as greater BMI, body mass and body fat percentage [42]. Interestingly, IRX3 KO mice expended more energy, particularly at night, due to a greater browning of white fatty tissue [42] and recent findings show a link between brown fat and muscle developmental precursor Myf5 [43] which may provide a mechanism for the observation of greater LM in *FTO* T allele carriers in our NT cohort. Moreover, using a transgenic mouse model (Rosa26^Enr-Irx3^) that disrupts IRX3 function whilst maintaining the genomic interaction between IRX3 and *FTO* (mimicking more accurately the human in vivo state than the aforementioned KO model), the authors showed retention of the KO model phenotype traits [42]. These *FTO*-IRX3 protein interactions suggest an explanation for the greater LM seen with the T allele carriers of the present study and possibly the association of the T allele with muscle power relative to body mass and its relationship with playing position in RU athletes (Tables 1, 2, and 4; Fig. 1).

The precise mechanisms of action of IRX3 in mammalian physiology are not fully understood, however the primary role of IRX3 in embryonic development and future actions in motor neuron restriction is relevant to this discussion. During neuronal development, IRX3 expression plays a key role in N-tubulin development and initiation of neuronal programming. High levels of IRX3 protein promote early tissue development resulting in a lack of cell differentiation [44]. Thus, it is possible that because the *FTO* T allele is associated with lower IRX3 expression, greater early motor neuron differentiation might subsequently lead to greater LM – as we observed (Table 1). As such, for predeterminant neuronal cells to differentiate into a progenitor motor neuron domain and subsequently motor neurons, it appears IRX3 must be repressed by the microRNA *mir-17-3p* in order for OLIG2 to regulate the development of ventral spinal motor neurons [45]. Thus, as the expression of OLIG2 increases, the yield of motor neurons increases in tandem [46]. Considering *FTO* T allele carriers have a lower embryonic expression of IRX3, T allele carriers may have a predisposition for greater LM through enhanced life-long motor neuron availability via OLIG2 expression and therefore, may be at an advantage for certain forms of athletic ability and associated performance phenotypes (Tables 2, 3 and 4; Fig. 1). This rationale and the present results may represent a small portion of the 85% heritability of adult muscle neuronal function [47].

Recent associations between *FTO* variants and IGF-1, specifically that serum IGF-1 levels were greater in T allele carriers [48], may provide a second mechanism to explain the observed genotype differences in LM (Table 1). It is well known that IGF-1 is upregulated as a consequence of mechanical load/exercise and plays an important role in the cellular development of muscle hypertrophy [49]. Hence, T allele carriers, who in the NT group had significantly greater LM, may experience upregulation of IGF-1 compared to AA genotype counterparts. Furthermore, serum IGF-1 levels have been positively associated with quadriceps torque [50] and explosive muscle power [51] in older men. These data provide a further potential basis for our observation that RU athletes who require greater muscle power relative to body mass (Table 4) show a greater frequency of the T allele than other playing positions (Table 2 and 3; Fig. 1).

We observed a lower frequency of the AA genotype in back three and centre playing positions (OR = 2.53; Table 3), although there was no difference between the entire rugby cohort and controls. That latter observation is similar to that we reported previously regarding another genetic variant (*ACTN3* rs1815739) where there was no difference between the entire rugby cohort and controls despite differences in genotype frequency according to playing position [22]. This again demonstrates the importance of defining athletes very carefully when conducting such comparisons. Global positioning system (GPS) data provide evidence for the relevance of our finding regarding *FTO* genotype in elite athletes. Jones et al. [35] showed that - at an elite competitive level - the back three and centre players express the greatest ‘instantaneous and accumulative demands for exercise’ (exertion index; EI) than all other athletes and spent more time at sprinting intensities. Thus, there is congruence between our dual observations of firstly greater LM in NT associated with the T allele, and secondly a greater frequency of the T allele in certain elite rugby athletes who rely on greater power outputs relative to body mass to be successful in those specific playing positions Table 3; [39].

These data suggest the relevance of the *FTO* rs9939609 T allele to muscle-related phenotypes and subsequently, athletic success. When considering the possible molecular mechanism from *FTO* via IRX3 to OLIG2 resulting in greater lifelong motor neuron availability, this may have implications for muscle size-related disorders such as sarcopenia and cachexia.

## Conclusions

The presented data show a novel dimension of *FTO* genetic variation in human physiology, by investigating in vivo muscle phenotypes in a healthy non-resistance trained population and relating those data to the extreme upper end of human physical performance – i.e. elite athletes. We show that the *FTO* rs9939609 protective T allele may be responsible for part of the inherited component of the inter-individual variability in LM. Furthermore, elite athletes who rely greatly on LM relative to total body mass for athletic success (RU back three and centre players, in this study) also seem more likely to carry a protective T allele, have higher peak muscle power output relative to body mass and are likely to selected for appropriate playing positions as a result of these and other phenotypes. It is possible that this association between the *FTO* rs9939609 SNP, via LM, and athletic success, could be a result of the interaction with IRX3 in embryonic development of motor neuron patterning and the IGF-1 muscle development pathway. The strengths of the presented paper are the two-layered study design (NT and elite athlete cohorts) and the combination of muscle functional phenotypes with case-control data. While the finding that *FTO* genotype differs among elite rugby playing positions is a new insight, *FTO* is only one of many variants (most others unknown) that contribute to this phenotype and as such should not be used for talent identification at this time. Replication is necessary for each cohort using comparable methods, and future experimental focus should be on the proposed biological pathways of these *FTO* associations with muscle phenotypes.

## Abbreviations

ACSA: Anatomical cross-sectional area
BMI: Body mass index
CMJ: Countermovement jump
DXA: Dual energy x-ray absorptiometry
FTO: Fat mass and obesity
IGF-1: Insulin-like growth factor 1
IRX3: Iroquois homeobox 3
LM: Leam mass
MVC_KE_: Maximal isometric knee extension
MVC_KF_: Maximal isometric knee flexion
NT: Non-resistance trained
OLIG2: Oligodendrocyte transcription factor
PCSA: Physiological cross-sectional area
PPO: Peak power output
QF: Quadriceps femoris
RL: Rugby league
RU: Rugby union
VL: Vastus laterals
*V*_VL_: VL muscle volume
